# Diffusion of a micronutrient home fortification program for infants and toddlers in a multi-ethnic population in rural western China

**DOI:** 10.1186/s12889-023-15746-0

**Published:** 2023-05-15

**Authors:** Ruixue Ye, Yuju Wu, Chang Sun, Qingzhi Wang, Yuping Mao, Huan Zhou, Hein Raat

**Affiliations:** 1 West China School of Public Health and West China Fourth Hospital, Sichuan University, Chengdu, Sichuan China; 2 Department of Communication Studies, College of Liberal Arts, California State University Long Beach, Long Beach, CA USA; 3 Department of Public Health, Erasmus University Medical Center, Rotterdam, Netherlands

**Keywords:** Micronutrient home fortification program, Complementary feeding, Multi-ethnic population, Diffusion of innovations theory, Adopter categories, Rural western China

## Abstract

**Background:**

The micronutrient home fortification programs contribute to the prevention of childhood anemia. WHO suggested applying culturally appropriate strategies to implement the micronutrient home fortification programs in various communities. However, there is little knowledge on evidence-based effective diffusion strategies of the micronutrient home fortification programs in multi-ethnic populations. This study aims to examine the diffusion of a micronutrient home fortification program with micronutrient powder (MNP) in a multi-ethnic population by investigating factors associated with being an ‘early’ or a ‘later’ adopter of MNP.

**Methods:**

We conducted a cross-sectional study in rural western China. Multistage sampling was used to select children’s caregivers in Han, Tibetan, and Yi ethnic communities (N = 570). The diffusion of innovations theory informed the data collection on caregivers’ decision process and was applied to classify participants into the MNP adopter categories of ‘leaders’, ‘followers’, ‘loungers’, and ‘laggards’. The ordered logistic regression model estimated the factors associated with the MNP adopter categories.

**Results:**

Caregivers from the Yi ethnic subgroup were likely to adopt MNP relatively late (AOR = 1.67; 95%CI = 1.09, 2.54) compared with Han and Tibetan ethnic subgroups. Caregivers with more knowledge regarding the MNP feeding method (AOR = 0.71; 95%CI = 0.52, 0.97) and those with stronger self-efficacy in adopting MNP (AOR = 0.85; 95%CI = 0.76, 0.96) were more likely to adopt MNP earlier than others. The following messages and channels also tend to make caregivers adopt MNP earlier: hearing that ‘MNP was free’ from villagers (AOR = 0.45; 95%CI = 0.20, 0.98), and learning ‘MNP feeding method’ from township doctors (AOR = 0.16; 95%CI = 0.06, 0.48).

**Conclusions:**

Disparities in adopting MNP existing among different ethnic groups require more effective diffusion strategies in disadvantaged minority ethnic groups. Enhancing self-efficacy in adopting MNP and knowledge on feeding method of MNP have the potential to make caregivers adopt MNP earlier. Peer networks and township doctors can be effective agencies to facilitate the diffusion and adoption of MNP.

**Supplementary Information:**

The online version contains supplementary material available at 10.1186/s12889-023-15746-0.

## Introduction

Children under five years old in low- and middle-income countries (LMICs) continue to suffer from micronutrient deficiencies, particularly iron-deficiency anemia, in the last decade [1, 2]. Early childhood anemia is often associated with insufficient childhood nutrition and may negatively affect health outcomes throughout an individual’s life [2, 3]. The World Health Organization (WHO) recommends that countries with high rates of childhood anemia (> 20%) distribute micronutrient powder (MNP) to meet children’s nutritional needs [4]. MNP programs have since been implemented internationally [5, 6]. Multiple studies have shown significant declines in childhood anemia after the introduction of the MNP program [5]; and the declines depend on the quality and quantity of the implementation of MNP [7].

In order to support the development of better implementation strategies, previous studies assessed the factors associated with the adoption of MNP by caregivers. A recent international meta-analysis indicated that the adoption level of MNP was associated with caregivers’ and children’s socio-demographic characteristics as well as caregiver’s knowledge and perceptions regarding feeding [8]. Other efforts focused on intervention strategies to improve the adoption of MNP. For example, two reviews described examples of how the MNP supply system may be improved [9, 10]. In a Bangladesh study, a household visit model was applied where female volunteer community health workers distributed MNP and provided infant and young children feeding counseling during home visits [11]. Despite these efforts, results from the evaluation of MNP programs in Nepal, Peru, South Mali, and western China have shown cases where the adoption of the MNP program was suboptimal; 20–40% of the caregivers from these studies never feed their child MNP during the appropriate age period [9–11].

The MNP program is a new preventive action that supports caregivers to provide their children MNP during a specific age period in order to decrease the possibility of malnutrition [5]. The MNP program is also a typical example of innovations according to the diffusion of innovation theory (DIT) [12]. DIT provides useful insights to understand the promotion strategies and adoption processes of MNP, which theory has been applied in the healthcare system and public health in China such as health technology (laparoscopic appendectomy during hospitalization) diffusion in hospital [13], the commercial sexual service seeking behavior diffusion among off-farm workers[14], purchase intention of iron-fortified soy [15]; and HPV vaccination adoption[16]. As indicated by the DIT [12], diffusion is the process by which information about an innovation is communicated through certain channels over time among the members of a social system. The DIT also distinguishes categories of individuals according to the phase in which they adopt an innovation, which helps define a segmentation of the target group of the intervention (i.e. the audience) [12]. Furthermore, the DIT has underscored that the diffusion strategy regarding an innovation should be compatible with the local culture to speed up the diffusion process [12]. In the same vein, the WHO specifically suggested that the implementation strategies of MNP programs, including the communication and delivery strategies, should be culturally appropriate for the target population [4].

In rural communities of western China, like other LMICs, research has found high rates of childhood anemia, with more than 50% of infants and toddlers suffering from iron-deficiency anemia [17, 18]. China’s government also implemented a MNP program (Named as “YingYangBao” in China) to decrease the high anemia rates in low socioeconomic status (SES) communities in rural areas [19, 20]. Local township and village doctors are in charge of delivering MNP and MNP information to local caregivers. Doctors typically distribute MNP and provide information about MNP to caregivers during routine children’s health checks at the township health centers or village clinics. In villages, if caregivers live far from the local clinic, doctors may make home visits [19, 21]. In rural western China, MNP programs have been implemented in diverse ethnic groups including the majority ethnic group Han and minority ethnic groups such as Tibetan, Yi, and Bai [19, 20, 22]. The culture of an ethnic group may shape the local values, communication patterns, and health behaviors [23], therefore culturally appropriate strategies have the potential to garner the effective implementation of the MNP program in ethnically diverse regions. However, previous studies mainly focused on the association of individual-level characteristics of children’s caregivers and the adoption of MNP [8], therefore, the diffusion patterns of MNP programs, and the factors that can facilitate adoption remain largely unknown in ethnically diverse populations in rural China.

In the present study, we use the survey data from a multi-ethnic community in order to explore the diffusion of the MNP program among caregivers with children aged 6–24 months. In particular, the aims of this study is to explore (1) in what patterns the MNP program is diffused among children’s caregivers who are ethnically Han, Tibetan, and Yi from rural western China; and (2) which factors are associated with being an ‘early’ or a ‘later’ adopter of MNP in this multi-ethnic population in rural western China.

## Methods

### Study design and participants

We conducted a cross-sectional survey in May 2019 in low-income, multi-ethnic areas of Sichuan Province in western China. Sichuan province is the largest western province with a population size of 91.22 million [24]. There were 32 counties enrolled in the MNP program in Sichuan province [25]. Of these 32 counties, 19 were primarily inhabited by Yi and Tibetan minorities [24]. This natural split between Han-majority and Yi- and Tibetan-minority counties with MNP implementation makes these areas ideal for conducting this study.

As shown in Appendix Figure S1, a multiple stage stratified sampling procedure was applied to recruit participants. First, we identified 32 MNP program rural counties in Sichuan Province. Among these 32 counties, we randomly selected two Han-majority ethnic counties, two Tibetan-minority ethnic counties, and two Yi-minority ethnic counties. Second, we randomly selected 6 townships within each sampled county. Townships that housed the county seat (typically more urbanized) were excluded. A total of 36 townships were enrolled. Third, 7 or 8 villages (depending on how many villages had a population over 800) were randomly selected from each township. A total of 283 villages were included in the study. Fourth, families with a child aged 6–24 months were enrolled from sampled villages [26]. A total of 1,449 caregivers provided informed consent and participated in the study. Out of 1,449 eligible caregivers, 374 caregivers were excluded from the population for analysis since they had experience using MNP before pregnancy or birth of their child (the sample deployed in the analysis only included the new adopters according to the DIT). Among the 1,075 caregivers without prior experience using MNP, 234 caregivers who had one or more missing values were excluded from the analysis. Moreover, among the 841 caregivers who had provided complete information, 271 eligible caregivers with non-valid information (such as ‘outliers’) were excluded from the analysis. The final sample for analysis consisted of 570 caregivers. For the result of the difference between enrolled and excluded subgroups, see Appendix Table S1.

### The development of survey questionnaire

Quantitative data were collected through survey interviews administered by trained enumerators using a structured survey questionnaire. The survey questionnaire developed based on our extensive literature review and two rounds of Delphi expert consultation. To start, we conducted a systematic literature search using databases such as PubMed, Web of Science, Embase, CAB Abstracts & MEDLINE (OVID), Cochrane Library, China National Knowledge Infrastructure (CNKI), Wanfang and VP. Our search terms included items such as micronutrient powders, micronutrient sprinkles, multiple micronutrients in powder, Ying Yang Bao, consumption, adherence, and so on. From our findings, we summarized the impact factors related to caregiver’s feeding practices using micronutrient powders. The target dimensions and items of the survey questionnaire were developed based on the above works.

After developing the survey questionnaire, 12 experts with backgrounds in maternal and child health, health behavior, nutrition and food hygiene, epidemiology and health statistics, social science, and health management, were invited to the two rounds of consultation. The experts evaluated the weight and importance of each dimension of the survey and whether it was reasonable. They also evaluated the importance, operability, and sensitivity of each item. The response rate was 100% in two rounds of consultation, Kendall’s W coefficient was 0.43 between the first and second consultation rounds.

Prior to implementation, we also conducted a pretest in non-sample villages using the finalized questionnaire. We analyzed the reliability and validity of the scale of the pretest survey, with Cronbach’s coefficients ranging from 0.70 to 0.83 and KMOs ranging from 0.66 to 0.76 for each dimension. We further refined the questions and field operation procedures to accommodate each of the study’s different local contexts. During this procedure, the changes/refinements included listing local names for the micronutrient powders, listing all communication channels available in each local setting, and implementing the locally used calendar in minority settings in order to determine each infant’s age with a high degree of accuracy. We carried out the reliability and validity of the scale of the formal survey, with Cronbach’s coefficients ranging from 0.65 to 0.69 and KMOs ranging from 0.67 to 0.76 for each dimension.

### Outcome variable

The outcome variable of this study is the MNP adopter category on the basis of DIT [12]. First, four survey items were applied to collect the information on caregivers’ decision-making process to adopt MNP: “When did you hear about MNP for the first time?”, “When did you decide to use MNP for the first time?”, “When did you receive MNP for the first time?”, and “When did you feed your child MNP for the first time?”. Second, the length of the decision period to adopt MNP (that is the time period from ‘hear about MNP for the first time’ to ‘feed MNP for the first time’) was calculated. Third, according to the DIT [12], individuals in a social system adopt an innovation in an over-time sequence rather than adopt it at the same time. Individuals can be divided into different adopter categories based on their innovation-decision period: the “innovators” are the first 2.5% of a group to adopt an innovation, the next 13.5% to adopt an innovation are labelled the “early adopters”, the next 34% of the adopters are called the “early majorities”, the following 34% of the group are the “late majorities”, and the last 16% are considered the “laggards” [12]. This categorical measure allowed us to distinguish five types of MNP adopter categories based on the length of the decision period. Because only a few caregivers were categorized as innovators, we combined the two groups (the innovators and the early adopters) into one group to get a more robust model when performing the analyses. Finally, four types of MNP adopter categories were assigned: (a) ‘leaders’, which includes those with the shortest decision period to adopt MNP; (b) ‘followers’, which includes the ‘early majorities’ to adopt MNP; (c) ‘loungers’, which includes the ‘late majorities’ to adopt MNP; and (d) ‘laggards’, which includes those with the longest decision period to adopt MNP.

### Independent variables

**General characteristics.** The researchers asked questions on the caregiver’s age, sex, ethnic background, educational level, occupation, and household assets. A household asset index was calculated using polychoric principal components analysis [27, 28] based on whether the household owned or had access to a water heater, washing machine, refrigerator, air conditioner, television, computer, motorcycle, car or truck. Caregivers were also asked about the child’s age, sex, preterm birth (the baby was born alive before 37 weeks of pregnancy duration[29]), low birth weight (the birth weight was lower than 2500 g[30]), and the child health status after birth which was rated on a 5-point Likert scale from 1 for “unhealthy” to 5 for “healthy”.

**Personality.** The Ten-Item Personality Inventory-Chinese version (TIPI-C)[31] was applied to describe the personality of the caregivers. TIPI-C included ten items which rated on a seven-point Likert scale from 1 for “disagree strongly” to 7 for “agree strongly”. The scale identified five personality dimensions: (a) extraversion; (b) agreeableness; (c) conscientiousness; (d) emotional stability; and (e) openness to experience.

**Cognition and self-efficacy regarding MNP.** Caregivers’ cognition regarding MNP was captured by the knowledge level of MNP as well as susceptibility and severity perception of children nutrition. The knowledge level of MNP was measured by three items: “What is the component of MNP?”, “What is the effect of MNP?”, and “What is the feeding method of MNP?”. These items were developed into three dummy variables with value 1 if the caregiver had correct knowledge. Susceptibility perception was measured by one item: “What was the probability of anemia to your child?”. Severity perception was assessed by one item: “Did you think that it was serious for your child if she or he had anemia problems?”. The responses to the susceptibility and severity perception items were rated on a 3-point Likert scale from 1 for “not at all” to 3 for “definitely”. The self-efficacy in adopting MNP was measured by one item: “Did you have confidence in solving all problems regarding adopting MNP?”. The responses to this item were rated on a 5-point Likert scale from 1 for “not at all” to 5 for “definitely”.

**Communication channel and message regarding MNP for the first time.** To assess the communication channel for receiving MNP information for the first time, researchers asked caregivers to select the main channel through which they initially received information about MNP, including: (a) family members or relatives; (b) villagers; (c) village doctors; and (d ) township doctors. Caregivers were also asked to select the main content of the message they received initially, including: (a) MNP was free; (b) the health benefits of MNP, such as preventing malnutrition and increasing immunity; and (c) MNP feeding method, such as using warm water to stir MNP and then to mix MNP with the child’s favorite food.

**Delivery pattern and distance regarding MNP.** The delivery pattern was measured by one item: “From where did you get or receive the MNP sachets most?” with responses including: (a) taking MNP from township health center; (b) taking MNP from village clinics; and (c) the health personnel or village cadre delivered MNP to home. The delivery distance to get or receive MNP was measured by one item: “How far is it from the MNP supply site to your home?” The researchers recorded the caregiver’s self-reported distance.

### Statistical analysis

“Chi-square tests”, “Fisher exact test”, and “analysis of variance” were used to assess differences between ethnic groups. The missing values were imputed using regression imputation, using the mi impute regress command in Stata for the imputation. Then, based on the DIT [12], diffusion curve of MNP program was plotted, and caregivers were classified into different types of the MNP adopter categories. Finally, ordered logistic regression models were constructed to assess the factors associated with the type of MNP adopter category. In the models, the MNP adopter category was the outcome variable; general characteristics, personality, cognition and self-efficacy regarding MNP, communication channel and message for the first time, delivery pattern and distance regarding MNP were independent variables. To explore the interaction effect of communication channels and communication messages on the MNP adopter categories, the interaction term between “communication channel” and “communication message” was incorporated in the models based on DIT. *P*-values below 0.05 were considered statistically significant. All statistical analysis were conducted using STATA Statistical Software Release 14.1.

### Ethical approval

This study was approved by the Sichuan University Medical Ethical Review Board (approval number: K2018103). Before conducting interviews, trained enumerators introduced the study aims, the process, the potential risks and benefits, the privacy measures that were taken, the rights and duties of the individual, and the contact for the study. Enumerators also presented each household with a standardized document that outlined the same information. All methods were carried out in accordance with relevant guidelines and regulations. The interviews were conducted after the children’s caregivers provided written informed consent for the children and their own involvement.

## Results

### Characteristics of the study participants

Caregivers’ average age was 38.40 years, 90.88% were female, 57.37% did not complete primary school education, 48.07% were stay-at-home caregivers, and 22.63% reported a low level of household assets. 25.61%, 16.84%, 16.67%, 17.54%, and 23.16% of caregivers reported a higher level of personality of extraversion, agreeableness, conscientiousness, emotional stability, and openness to experience, respectively. Regarding child’s characteristics, the average age was 14.20 months, 52.28% were boys, 6.41% were born preterm, 14.01% had low birth weight, and the average score of self-reported child health status after birth was 4.50. Regarding cognition and self-efficacy regarding MNP, 11.58%, 27.19%, and 56.49% of caregivers had knowledge of the MNP component, MNP effect, and MNP feeding method, respectively. 22.81% and 22.46% of caregivers had higher levels of susceptibility and severity cognition of child anemia, respectively. 19.12% had higher levels of self-efficacy in adopting MNP. Regarding communication channel and message regarding MNP, 54.91% received the MNP message from village doctors and 58.42% received the message on the health benefits of MNP for the first time. Regarding delivery pattern and distance of MNP, 52.11% had taken MNP from the township health center, the distance from home to supply site of MNP was average 4.20 km. Compared with Tibetan and Yi groups, caregivers from the Han group were older, more often female, with higher educational and household asset levels, with more personality of extraversion and conscientiousness, and emotional stability, and less often with a child with preterm birth or low birth weight. Caregivers from the Han group also had higher levels of cognition and self-efficacy regarding MNP. Compared with Han groups, caregivers from the Tibetan and Yi group more often receive MNP messages from village doctors and get the message on health benefits of MNP, they also more often take MNP from village clinics or received MNP at home (all *p* < 0.05) (Table 1).

**Table 1:** Characteristics of the study participants in rural western China, 2019

Characteristics	Total	Han ethnic group	Tibetan ethnic group	Yi ethnic group	*p*-value ^a^
**Caregiver’s characteristics**					
Age (years)	38.40	42.00	39.90	33.20	**< 0.001**
Female (%)	90.88	96.89	80.56	91.54	**< 0.001**
Education level (%)					**< 0.001**
Did not complete primary school	57.37	31.56	57.64	86.07	
Primary school	12.98	16.89	13.89	7.96	
Junior school	16.32	31.56	11.81	2.49	
High school or above	13.33	20.00	16.67	3.48	
Occupation (%)					**< 0.001**
Stay-at-home caregivers	48.07	69.33	56.94	17.91	
Farmer/nomadic herder	30.53	6.67	9.72	72.14	
Other ^b^	21.40	24.00	33.33	9.95	
Household assets level (%) ^c^					**< 0.001**
Low level	22.63	0.89	4.86	59.70	
Lower than mean	28.25	20.00	27.78	37.81	
Higher than mean	33.16	45.78	56.94	1.99	
High level	15.96	33.33	10.42	0.50	
**Personality of caregivers** ^d^					
Extraversion (%)					**0.04**
Low level	20.35	17.78	29.86	16.42	
Lower than mean	30.88	30.22	27.78	33.83	
Higher than mean	23.16	23.56	18.75	25.87	
High level	25.61	28.44	23.61	23.88	
Agreeableness (%)					**0.01**
Low level	24.91	27.11	31.25	17.91	
Lower than mean	25.09	27.11	20.14	26.37	
Higher than mean	33.16	35.11	32.64	31.34	
High level	16.84	10.67	15.97	24.38	
Conscientiousness (%)					**0.02**
Low level	18.07	14.67	27.08	15.42	
Lower than mean	38.60	36.44	34.03	44.28	
Higher than mean	26.67	29.78	25.69	23.88	
High level	16.67	19.11	13.19	16.42	
Emotional stability (%)					0.37
Low level	24.39	23.56	28.47	22.39	
Lower than mean	32.11	29.33	34.72	33.33	
Higher than mean	25.96	28.89	18.75	27.86	
High level	17.54	18.22	18.06	16.42	
Openness to experience (%)					**< 0.001**
Low level	15.61	19.11	21.53	7.46	
Lower than mean	37.72	40.44	23.61	44.78	
Higher than mean	23.51	22.22	23.61	24.88	
High level	23.16	18.22	31.25	22.89	
**Child’s characteristics**					
Age (months)	14.20	14.50	13.80	14.20	0.40
Boy (%)	52.28	51.11	51.39	54.23	0.79
Preterm birth (%) ^e^	6.41	6.94	10.53	3.05	0.02
Low birth weight (%) ^f^	14.01	5.45	9.03	27.00	**< 0.001**
Self-reported child health status after birth, average score ^g^	4.50	4.40	4.50	4.70	**0.01**
**Cognition and self-efficacy regarding MNP**					
Have knowledge of MNP component (%)	11.58	20.89	9.03	2.99	**< 0.001**
Have knowledge of MNP effect (%)	27.19	32.44	21.53	25.37	0.06
Have knowledge of MNP feeding method (%)	56.49	67.11	58.33	43.28	**< 0.001**
Susceptibility of child anemia (%)					**< 0.001**
Not at all	41.75	22.22	50.00	57.71	
Probably	35.44	56.44	27.78	17.41	
Definitely	22.81	21.33	22.22	24.88	
Severity of child anemia (%)					**< 0.001**
Not at all	59.47	40.00	63.89	78.11	
Probably	18.07	30.67	15.28	5.97	
Definitely	22.46	29.33	20.83	15.92	
Self-efficacy in adopting MNP (%)					**< 0.001**
Not at all	26.32	13.33	28.47	39.30	
Probably not	10.53	6.67	19.44	8.46	
Neutral	15.79	19.11	15.28	12.44	
Probably	28.25	37.78	25.69	19.40	
Definitely	19.12	23.11	11.11	20.40	
**Communication channel and message regarding MNP for the first time**					
Communication channel for the first time (%)					**< 0.001**
Family members or relatives	9.65	16.00	6.94	4.48	
Villagers	8.42	13.33	1.39	7.96	
Village doctors	54.91	36.44	58.33	73.13	
Township doctors	27.02	34.22	33.33	14.43	
Communication message for the first time (%)					**0.02**
MNP was free	32.63	37.33	22.22	34.83	
Health benefits of MNP	58.42	52.89	70.14	56.22	
MNP feeding methods	8.95	9.78	7.64	8.96	
**Delivery pattern and distance of MNP**					
Delivery pattern regarding MNP (%)					**< 0.001**
Taking MNP from township health center	52.11	88.00	43.75	17.91	
Taking MNP from village clinics	33.86	7.56	39.58	59.20	
The health personnel or village cadre delivered MNP to home	14.04	4.44	16.67	22.89	
Distance to supply site (km)	4.20	3.00	6.70	3.80	**< 0.001**

^a.^ Chi-square tests of independence between ethnic groups were conducted. Boldface indicates statistical significance (p < 0.05)

^b.^ Other occupations include off-farm part-time jobs and self-employment

^c.^ A household asset index was calculated using polychoric principal components analysis[27, 28] based on whether the household owned or had access to a water heater, washing machine, refrigerator, air conditioner, television, computer, motorcycle, car or truck

^d.^ The Big Five personality traits scale, developed from the 1980s onwards in psychological trait theory, was applied to test the personality of caregivers in our study. The scale identified five personality dimensions: extraversion, agreeableness, openness to experience, conscientiousness, and emotional stability

^e.^ Preterm birth means the infant was born alive before 37 weeks of pregnancy duration.[29]

^f.^ Low birth weight means the birth weight was lower than 2500 g.[30]

^g.^ Caregivers were asked about child health status after birth, the responses were rated on a 5-point Likert scale that ranged from 1 for ‘unhealthy’ to 5 for ‘healthy’

### The diffusion pattern of the MNP program

Caregivers heard about MNP at mean child age 0.8 months, decided to use MNP at mean child age 7.8 months, received the MNP at mean child age 8.3 months, and fed the child MNP first time at mean child age 8.9 months. Caregivers of Han group heard about MNP, decided to use MNP, received MNP, and fed the child MNP earlier than caregivers in the Tibetan and Yi groups (*p* < 0.05). The decision period to adopt MNP in the whole study population was on average 19.6 months; among the three ethnic groups, Han group was 18.5 months, Tibetan group was 18.7 months, and Yi group was 21.6 months (*p* < 0.001). For the details of the decision process of adopting MNP, see the Appendix Table S2.

**Fig. 1:**
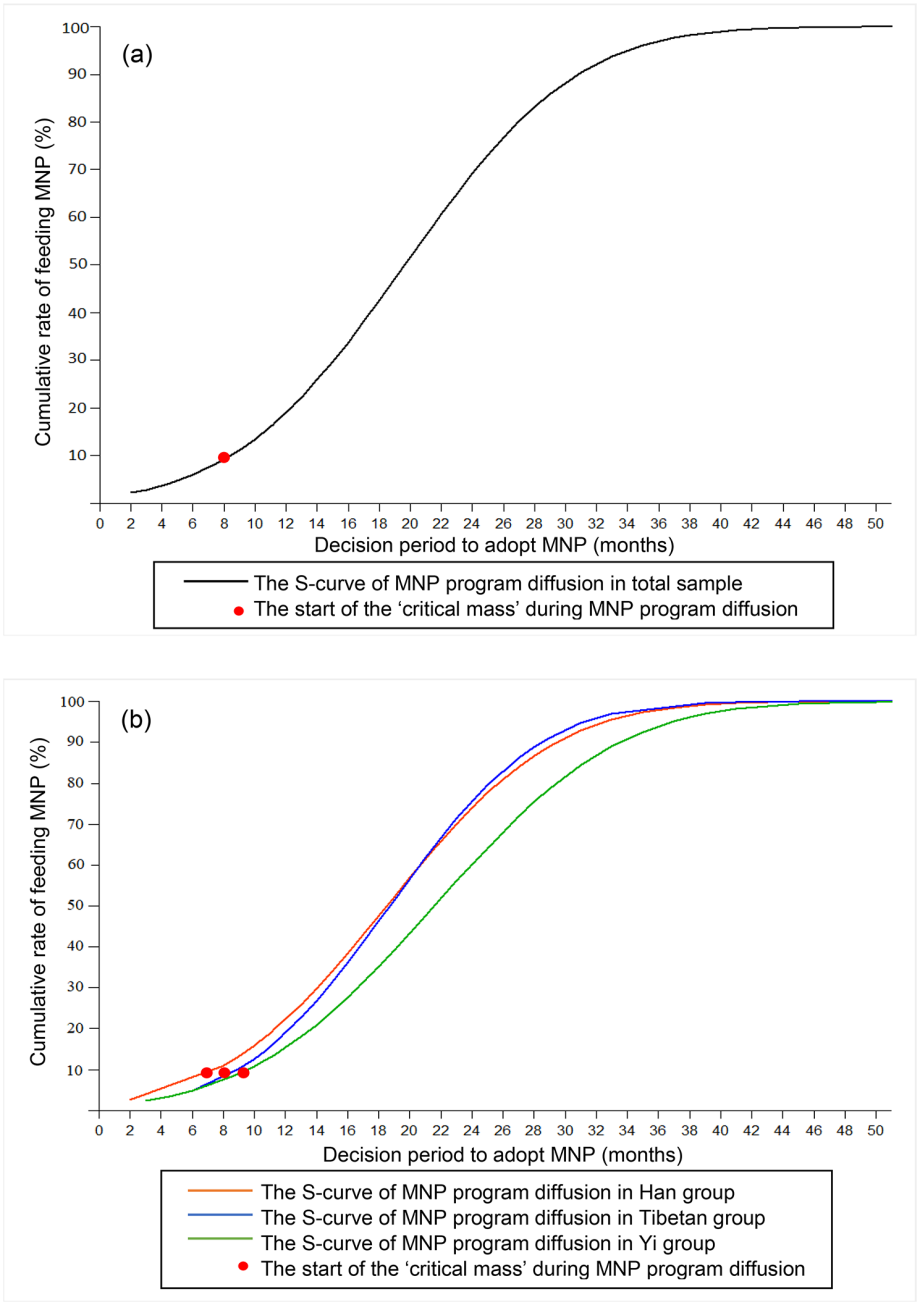
(**a**) Diffusion curve of MNP program among caregivers with children aged 6–24 months old in rural western China; (**b**) Diffusion curve of MNP program among caregivers with children aged 6–24 months old in Han, Tibetan, and Yi ethnic group from rural western China

Figure 1 shows the diffusion curve of MNP program in the study population. In Fig. 1a, the horizontal scale indicates the decision period (in months) to adopt MNP, and the vertical scale indicates the cumulative rate of feeding MNP in the whole study population (over time). Approximately 10% of the caregivers fed the child MNP within 8 months after they heard about MNP for the first time. As indicated in Figs. 1a, 8 months is the point of ‘critical mass’, where the ‘diffusion started to take off’. At this point, the slope in the mid-section of the curve becomes relatively steeper (i.e., the feed rate accelerates) as caregivers ‘jump on the bandwagon’. Finally, the curve plateaus when the increase regarding the diffusion slowed down and eventually approached a horizontal line when approximately 90% of caregivers fed the child MNP. It is worth noting that differences exist among Han, Tibetan, and Yi groups in the diffusion curves. As shown in Fig. 1b, Han, Tibetan, and Yi ethnic groups respectively had 7, 8, and 9 as the “critical mass” points when the ‘diffusion started to take off’; Han and Tibetan ethnic groups had a higher slope of curves than the Yi ethnic group during the MNP diffusion.

**Fig. 2:**
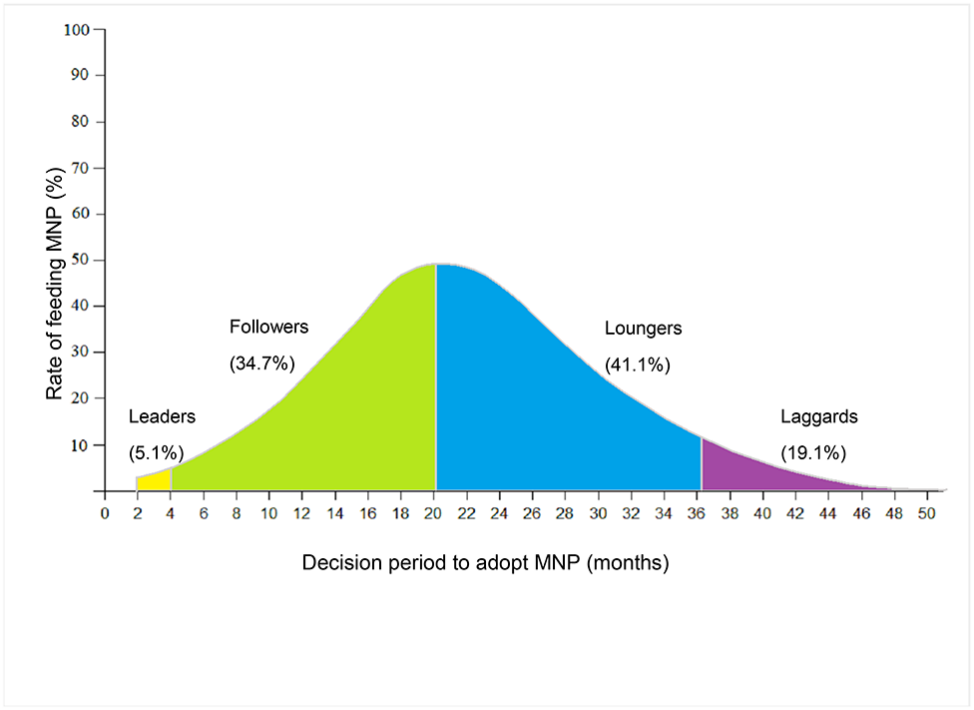
MNP adopter categories among caregivers with children aged 6–24 months old in rural western China. Notes: Based on the DIT, the frequency distribution of the number of adopters in different decision periods to adopt MNP can be visualized, which approaches a normal, bell-shaped curve. Under the bell-shaped curve, four types of MNP adopters were identified. The most left yellow area was ‘leaders’; ‘followers’ were recognized in the green area; ‘loungers’ were in the blue area; and the most right purple area was identified as ‘laggards’ during MNP program diffusion

Figure 2 indicates the frequency distribution of the number of adopters in the different decision period to adopt MNP, which approaches a normal, bell-shaped curve. Based on DIT, participants were categorized into four types of MNP adopters shown in Fig. 2: 5.1% (29/570) caregivers were considered to be ‘leaders’ (the yellow area); the next 34.7% (198/570) were considered to be ‘followers’ (the green area); 41.1% (234/570) were considered to be ‘loungers’ (the blue area); and 19.1% (109/570) were considered to be ‘laggards’ (the purple area).

### Factors associated with the MNP adopter category

Table 2 shows the factors associated with different types of MNP adopters based on the ordered logistic regression analysis. After controlled the covariates (such as the education level, extraversion personality, and knowledge regarding the MNP component, etc.), belonging to the Yi group (adjusted odds ratio[AOR] = 1.67; 95% confidence interval[CI] = 1.09, 2.54), having knowledge regarding the MNP feeding method (AOR = 0.71; 95%CI = 0.52, 0.97), a high level of self-efficacy in adopting MNP (AOR = 0.85; 95%CI = 0.76, 0.96), and the interaction terms of ‘villagers * free’ (AOR = 0.45; 95%CI = 0.20, 0.98), and ‘township doctors * method’ (AOR = 0.16; 95%CI = 0.06, 0.48) were associated with the MNP adopter category. For the results of the univariate analyze, refer to the Appendix Table S3. For the results of the subgroup analysis among Han, Tibetan, and Yi ethnic groups, refer to the Appendix Table S4.

**Table 2:** Ordered logistic regression model of the associated factors of MNP adopter category among caregivers with children aged 6–24 months in rural western China, 2019

Characteristics	MNP adopter category ^a,b^		
	AOR	95% CI	
**Caregiver’s characteristics**			
Ethnic background			
Han ethnic group	Ref.		
Tibetan group	1.14	0.75	1.74
Yi ethnic group	1.67*	**1.09**	**2.54**
Education level	0.96	0.81	1.14
**The personality of caregivers** ^c^			
Extraversion	0.92	0.80	1.07
**Cognition and self-efficacy regarding MNP**			
Knowledge regarding the MNP component	0.79	0.48	1.3
Knowledge regarding the MNP effect	0.95	0.67	1.36
Knowledge regarding the MNP feeding method	0.71*	**0.52**	**0.97**
Susceptibility perception regarding childhood anemia	1.09	0.88	1.36
Self-efficacy in adopting MNP	0.85**	**0.76**	**0.96**
**Communication channel and message regarding MNP for the first time**			
Family members or relatives*free	Ref.		
Family members or relatives*benefit	0.89	0.29	2.67
Family members or relatives*method	0.91	0.18	4.64
Villagers*free	0.45*	**0.20**	**0.98**
Villagers*benefit	0.71	0.25	2.03
Villagers*method	0.23	0.02	2.66
Village doctors*free	0.64	0.26	1.61
Village doctors*benefit	0.55	0.24	1.27
Village doctors*method	0.27	0.07	1.05
Township doctors*free	0.53	0.24	1.19
Township doctors*benefit	0.50	0.20	1.27
Township doctors*method	0.16**	**0.06**	**0.48**

Note: AOR = adjusted odds ratio; CI = confidence interval. Boldface indicates statistical significance (*p < 0.05, **p < 0.01, ***p < 0.001)

^a.^ In the ordered logistic regression model, the MNP adopter category was regarded as the outcome variable, the value assignment of MNP adopter category was: 1 = leaders; 2 = followers; 3 = loungers; 4 = laggards

^b.^ In the ordered logistic regression model, the p-value of variables less than 0.10 from univariate analysis were enrolled as the independent variables, including ethnic background, education level, the personality of extraversion, knowledge regarding MNP component, knowledge regarding MNP effect, knowledge regarding MNP method, susceptibility of child anemia, have confidence in solving all problems on adopting MNP, and interaction between communication channel and message regarding MNP for the first time

^c.^ The Big Five personality traits scale, developed from the 1980s onwards in psychological trait theory, was applied to test the personality of caregivers in our study. The scale identified five personality dimensions: extraversion, agreeableness, openness to experience, conscientiousness, and emotional stability

## Discussion

Our study examined the diffusion of the MNP program, a preventive innovation, in vulnerable populations in low income multi-ethnic (Han, Tibetan, and Yi) communities. The study showed that the MNP program diffused slowly; caregivers’ ethnic background, knowledge regarding MNP feeding method, self-efficacy in adopting MNP, and communication patterns regarding MNP, were associated with the MNP adopter category.

Although the MNP program has been found to be safe and effective [5], the study showed that the diffusion of MNP program in rural western China was suboptimal. According to the guidelines of the MNP program, when the child’s mother is pregnant, the target family develops awareness regarding the importance of using MNP. The next step is that the family receives MNP from local health providers and feeds MNP to the child from 6 months onwards [22]. Our results showed that across the three ethnic groups, not all caregivers adhered to this guideline. Relatively often, the knowledge regarding the use of MNP was not present during pregnancy, but after the child was born. Also, relatively few caregivers decided to use MNP once they heard about it. The study illustrates a relatively long time period from caregivers’ decisions to use MNP to the subsequent actions of receiving and actually feeding the child MNP. The diffusion of the MNP program is the process that caregivers gradually adopt MNP. Previous studies have shown that the adoption of using MNP is generally suboptimal. This may impede the diffusion of the MNP program in the target population. For example, two studies from China indicated that 20.0% and 42.1% of caregivers did not adhere to feeding MNP children[32, 33]. Studies from South Mali and China found that 18.5% and 20.0% of caregivers never provided MNP to children, respectively [6, 34]. One study from Peru found that 24.1% of caregivers did not receive MNP [35]. The findings could be understood in light of the DIT. According to the DIT, most types of innovations require a relatively long time period to be accepted by the target audience. Especially the preventive innovations (like the MNP program in our study) are likely to be diffused slowly due to ‘delayed rewards’ that the audience experienced from the adoption of the innovation [12].

One important finding of this study is that caregivers belonging to the Yi ethnic group were more likely to adopt MNP late. Earlier studies confirmed the presence of ethnic differences regarding the adoption of MNP in China [32, 33]. This may be partly explained by differences in SES (including education level, occupation, and household assets) [33, 36], language barriers (Mandarin is the primary language used in mass media, which is the common language among the Han group) [33, 37], and differences in communication channels and messages regarding MNP information across Han, Tibetan, and Yi communities [26]. Therefore we recommend culturally-adapted strategies to speed up the diffusion process in the Yi ethnic community.

Beyond ethnic background, our study also identified the following factors associated with the diffusion process of MNP: caregivers’ knowledge of how and when MNP should be provided (i.e. ‘knowledge regarding the MNP feeding methods’), and caregivers’ attitudes towards the use of MNP (i.e. ‘self-efficacy in adopting MNP’). Specifically, caregivers were more likely to adopt MNP early when they had a correct understanding of using MNP; such caregivers also reported a stronger self-efficacy in adopting MNP. As the DIT explains, the ‘early adopters’ usually have more knowledge regarding the innovation than ‘late adopters’ [12]. Also, individual are more likely to adopt an innovation if they believe they can solve potential problems by adopting the innovation; these individuals tend to have a higher level of self-efficacy regarding the adoption of the innovation [38]. This was confirmed by a recent review that showed a higher level of knowledge and efficacy are linked with better practices regarding the adoption of MNP [8]. Based on the similar pattern found in our study, we suggest providing more support for and having more effective communication with caregivers to increase their awareness, knowledge, and skills of MNP.

Accordingly, we further found that the communication channels used by the caregivers and the content of the messages were predicative of an early adoption of MNP. When the caregivers received information from villagers, and in the case that villagers told that the MNP was provided free of cost (i.e. the interaction term of ‘villagers*free’ in this study), there was a relatively higher chance for the caregiver to adopt MNP early. Caregivers who obtained information on MNP feeding methods from township doctors (i.e. the interaction term of ‘township doctors*method’ in this study) were more likely to be early adopters of MNP. In line with the DIT, it can be expected that ‘incentives’ such as the free-of-cost use of the innovation can promote early adoption, at least until a ‘critical mass’ is reached [12]. Similarly, communicate the innovation among near peers who was regarded as the reliable channels for the audience, can be expected to ‘energize’ the diffusion process [12]. A previous study also indicated that despite the presence of obvious ‘advantages’, the implementation of innovations may fail due to the complex nature of the innovations [12]. Our results showed that most caregivers received the information on free-of-cost MNP (32.63%) and about the health benefits of MNP (58.42%) when they heard about MNP for the first time. Only a few caregivers received information on MNP feeding methods(8.95%). The DIT indicates that technical information (such as the MNP feeding methods in our study) is essential to the decision-making process. This type of information would encourage the audience to adopt the innovation [12]. Thus, it is meaningful to design communication strategies to spread technical information about the MNP feeding methods to caregivers. The agent who influences audiences’ innovation-decisions can facilitate the diffusion of innovation [12]. As one of the agent of the MNP program innovation, the local health personnel (such as township doctors), therefore can help to overcome the diffusion barriers such as the complex information and practice of MNP by teaching caregivers how to use MNP.

This study makes two key contributions to the literature. First, our findings offer empirical evidence to apply audience-segmentation strategies to accelerate the diffusion of MNP programs in rural China. Second, this is the first study to apply the DIT to examine the diffusion of the MNP program. The application of DIT provides a group-level perspective to explore the diffusion of the preventive innovations in LMICs.

The findings of this study should be interpreted with caution because the data were self-reported by the caregivers. First, there is a possibility of recall bias, which is one of the common weaknesses of research on diffusion processes. Future research could consider research designs such as longitudinal panel study that can better ensure the validity of data on the diffusion process. Second, even though interpersonal communication was the main channel in low income and multi-ethnic communities, mass media channels may help to distribute MNP knowledge among caregivers who have the ability to understand the information. Future studies should consider to assess the role of mass media channels in the study. Third, delayed delivery of the MNP from the doctor’s side may cause a delay in the decision-making process to adopt MNP among caregivers. Even though we had enrolled the delivery mode and delivery distance of the MNP into the model, in our study the delivery time of the MNP (e.g. delivery frequency) was not enrolled into the model. In order to examine the role of delivery mode in MNP adopter categories thoroughly, future studies should enroll the delivery time of MNP in the survey questionnaire and data analysis. Fourth, in order to provide a more in-depth understanding of how culture influences caregivers’ decisions to adopt MNP, future research using qualitative approaches should be considered.

## Conclusions

This study was performed in a relatively neglected and vulnerable population. It examined how the MNP program was diffused, and identified the factors were associated with being an ‘early’ or a ‘later’ adopter regarding the use of MNP in rural western China. Our findings show that better support for children’s caregivers could positively influence their adoption of MNP in ethnically diverse regions. Enhancing caregivers’ self-efficacy and knowledge on MNP could potentially make the diffusion and implementation of MNP more effectively by bridging the knowledge disparities across various groups. To successfully implement the MNP program in low income and multi-ethnic communities, it is important to promote and spread the free-of-cost advantage of the MNP via near-peer networks. Findings of this study also suggest encouraging more well-trained township doctors to deliver technical information about the MNP feeding methods to caregivers.

## Electronic supplementary material

Below is the link to the electronic supplementary material.

Supplementary Material 1

## Data Availability

The datasets analyzed for the current study are not publicly available due to ethical restrictions related to the consent given by participants at the time of study commencement. An ethically compliant dataset may be made available by the corresponding author on reasonable request and upon approval by the Sichuan University Medical Ethical Review Board. Requests to access the datasets should be directed to zhouhuan@scu.edu.cn.

## Abbreviations

DIT: Diffusion of Innovation Theory
LMICs: Low- and Middle-Income Countries
MNP: Micronutrient Powder
SES: Socioeconomic Status
TIPI-C: Ten-Item Personality Inventory-Chinese version
