# Supplementary material for: Diffusion of a micronutrient home fortification program for infants and toddlers in a multi-ethnic population in rural western China
Source: BMC Public Health. 2023 May 15;23:889. doi: 10.1186/s12889-023-15746-0 (PMC10184398; doi:10.1186/s12889-023-15746-0)
Supplement: Supplementary file 1 — Supplementary Material 1 [file 12889_2023_15746_MOESM1_ESM.docx]

**Additional files**


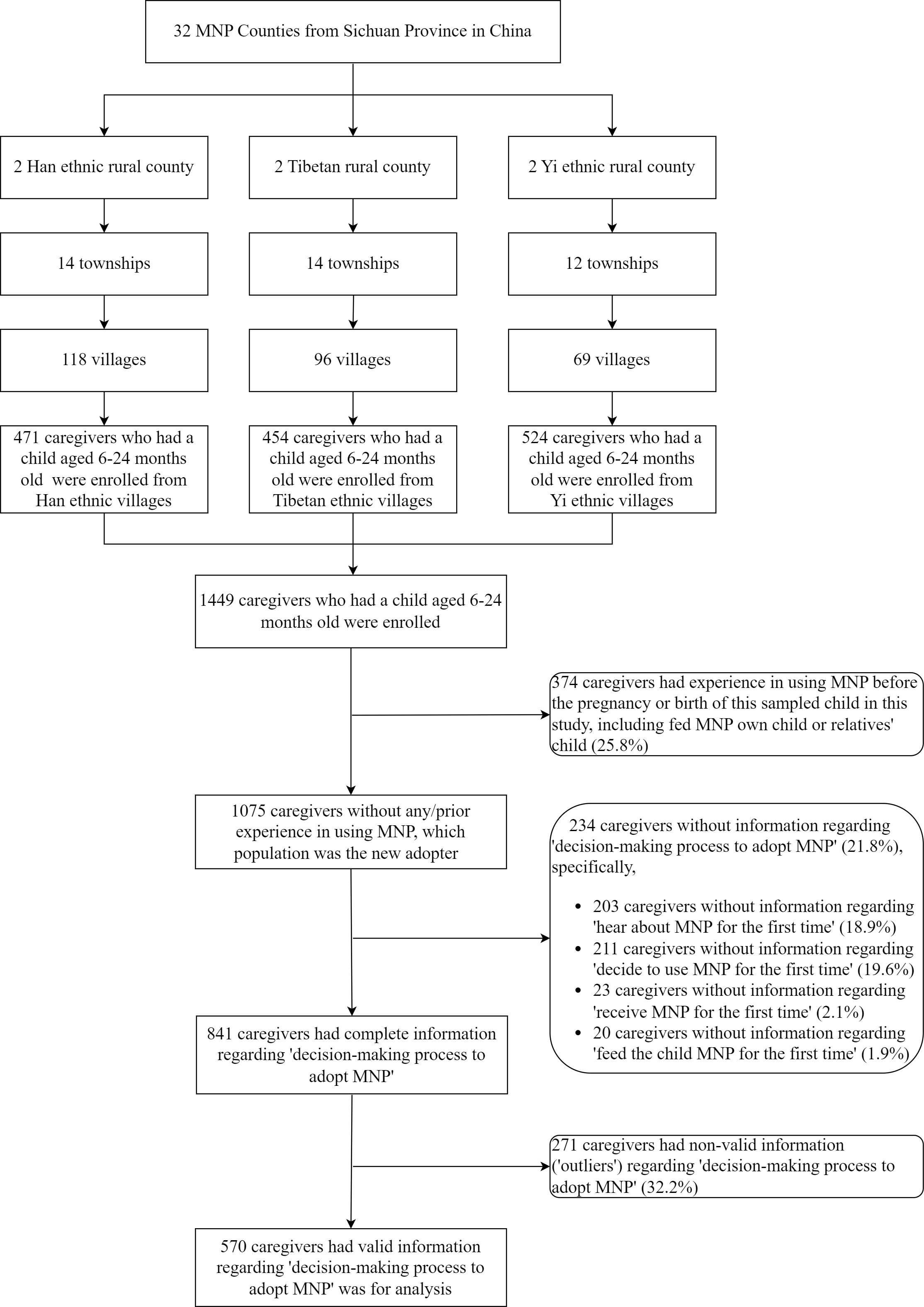


**Appendix Figure S1.** Flowchart of sampling among caregivers with children aged 6-24 months old in rural western China

Note: completed information means that caregivers had not the missing value regarding ‘decision process to adopt MNP’; valid information means that caregivers had not the outlier value regarding ‘decision process to adopt MNP’.

| **Appendix Table S1.** The result of the difference between enrolled and excluded subgroups among caregivers with child aged 6-24 months old in rural western China, 2019 | | | | | | | | | |
| --- | --- | --- | --- | --- | --- | --- | --- | --- | --- |
| **Characteristics** | **Without experience** | **Have experience** | ***p*-value** ^a^ | **Without missing value** | **Have missing value** | ***p*-value** ^a^ | **With valid value** | **Have valid value** | ***p*-value** ^a^ |
| **Caregivers' characteristics** |  |  |  |  |  |  |  |  |  |
| Age (years) | 37.70 | 37.00 | 0.39 | 37.9 | 37.2 | 0.45 | 38.3 | 36.9 | 0.17 |
| Female (%) | 91.61 | 93.33 | 0.29 | 8.59 | 7.69 | 0.66 | 90.73 | 92.73 | 0.33 |
| Ethnic background (%) |  |  | 0.29 |  |  | **<0.001** |  |  | **0.01** |
| Han ethnic | 32.66 | 31.20 |  | 35.68 | 22.22 |  | 39.51 | 27.64 |  |
| Tibetan | 30.07 | 34.40 |  | 28.04 | 38.03 |  | 25.17 | 33.82 |  |
| Yi ethnic | 37.27 | 34.40 |  | 36.28 | 39.74 |  | 35.31 | 38.55 | |
| Education level (%) |  |  | 0.94 |  |  | 0.09 |  |  | 0.64 |
| Did not complete  primary school | 58.95 | 59.20 |  | 57.28 | 64.53 |  | 57.34 | 57.45 |  |
| Primary school | 13.56 | 13.87 |  | 14.08 | 11.11 |  | 13.29 | 16.00 |  |
| Junior school | 14.30 | 13.07 |  | 15.51 | 10.26 |  | 16.26 | 13.82 |  |
| High school or above | 13.19 | 13.87 |  | 13.13 | 14.10 |  | 13.11 | 12.73 |  |
| Occupation (%) |  |  | 0.07 |  |  | **<0.001** |  |  | 0.50 |
| Stay-at-home caregivers | 46.49 | 44.00 |  | 49.88 | 33.33 |  | 48.78 | 53.09 |  |
| Farmer/nomadic herder | 27.58 | 24.00 |  | 29.47 | 21.37 |  | 30.07 | 27.64 |  |
| Other ^b^ | 25.92 | 32.00 |  | 20.64 | 45.30 | | 21.15 | 19.27 |  |

| **Continued Appendix Table S1.** | | | | | | | | | |
| --- | --- | --- | --- | --- | --- | --- | --- | --- | --- |
| **Characteristics** | **Without experience** | **Have experience** | ***p*-value** ^a^ | **Without missing value** | **Have missing value** | ***p*-value** ^a^ | **With valid value** | **Have non-valid value** | ***p*-value** ^a^ |
| Household assets level (%) |  |  | 0.24 |  |  | **0.04** |  |  | 0.36 |
| Low level | 25.92 | 25.60 |  | 24.22 | 31.62 |  | 22.55 | 28.00 |  |
| Lower than mean | 28.14 | 25.07 |  | 27.80 | 29.06 |  | 28.32 | 26.55 |  |
| Higher than mean | 30.35 | 35.73 |  | 32.22 | 23.93 |  | 33.39 | 29.82 |  |
| High level | 15.59 | 13.60 |  | 15.75 | 15.38 |  | 15.73 | 15.64 |  |
| **Child's characteristics** |  |  |  |  |  |  |  |  |  |
| Age (months) | 14.1 | 14.8 | 0.05 | 14.2 | 14.3 | 0.66 | 14.2 | 13.9 | 0.50 |
| Boy (%) | 52.31 | 52.00 | 0.92 | 52.39 | 52.14 | 0.95 | 51.75 | 53.45 | 0.64 |
| Preterm birth (%) ^c^ | 8.01 | 12.26 | **0.02** | 6.54 | 13.22 | **0.01** | 6.38 | 6.67 | 0.87 |
| Low birth weight (%) ^d^ | 16.70 | 16.62 | 0.97 | 15.14 | 20.94 | **0.03** | 14.13 | 17.45 | 0.21 |
| Self-reported child health  status after birth,  average score ^e^ | 4.6 | 4.5 | 0.29 | 4.5 | 4.6 | 0.07 | 4.5 | 4.5 | 0.84 |
| **Number of participants** | 1075 | 374 |  | 841 | 234 |  | 570 | 271 |  |
| 1. Chi-square tests of independence between subgroups were conducted, for which *p*-values below 0.05 were considered statistically significant. 2. Other occupations include off-farm part-time jobs and self-employment. 3. Preterm birth is defined as the baby was born alive before 37 weeks of pregnancy duration. 4. Low birth weight means the birth weight was lower than 2500 grams. 5. Caregivers were asked about child health status after birth, the responses were rated on a 5-point Likert scale that ranged from 1 for ‘unhealthy’ to 5 for ‘healthy’. | | | | | | | | | |

**Appendix.** The calculation of innovation-decision period to adopt MNP

The innovation-decision period to adopt MNP in this study was defined as the duration from the time points between ‘hear about MNP for the first time’, to ‘decide to use MNP for the first time’, to ‘received MNP for the first time’, and until to ‘feed the child MNP for the first time’. The calculation of this period (time length) could be divided into two episodes.

For the first episode:

The first episode was the duration between ‘hear about MNP for the first time’ and ‘decide to use MNP for the first time’. In order to obtain a reasonable time length, the time point of ‘hear about MNP for the first time’ had been adjusted in the calculation. Specifically, according to the guidelines of the MNP program, the target audience, pregnant women (child age was from -9 to 0 months old) and caregivers with child (child age was from 0 to 24 months old), should be received the MNP message from the local township health center and village clinics. However, for some caregivers, it would be possible to hear about MNP before pregnancy (that is before the child’s age was -9), they actually had the ‘waiting time’ to know MNP. For this population, the ‘reward’ was given when calculated, since they had known MNP in advance. On contrary, it would be possible that some caregivers heard about MNP after pregnancy (that is after the child’s age was -9), they had the ‘lagging time’ to know MNP, the ‘punishment’ was given to this population when calculated since they were delayed to know MNP. So the child age of ‘-9 months old’ was used to adjust the time point of ‘hear about MNP for the first time’ in the calculation (as the following formula 1).

The duration between ‘hear about MNP for the first time’ to ‘decide to use MNP for the first time’ = (decide time point – heard about time point) + [heard about time point-(-9)] = decide time point + 9 (1)

For the second episode:

The second episode was the duration between ‘receive MNP for the first time’ and ‘feed the child MNP for the first time’. In order to get a reasonable time length, the time point of ‘receive MNP for the first time’ had been adjusted in the calculation as well. In specific, according to the guidelines of the MNP program, caregivers should receive MNP from the local township health center or village clinics when their child was 6 months old [1,2]. However, some caregivers would receive MNP after their child was 6 months old, they actually had the ‘lagging time’ to receive MNP. For this population, the ‘punishment’ was given in calculation since they had received MNP after the recommended time. So the child age of ‘6 months old’ was used to adjust the time point of ‘receive MNP for the first time’ in the calculation (as the following formula 2).

Duration between ‘receive MNP for the first time’ to ‘feed the child MNP for the first time’ = (feed time point - receive time point) + (receive time point - 6) = (feed time point - 6) (2)

Finally, the sum of the first episode (from ‘hear about MNP for the first time’ to ‘decide to use MNP for the first time’) and the second episode (from ‘receive MNP for the first time’ to ‘feed the child MNP for the first time’) was regarded as the innovation-decision period to adopt MNP among caregivers in our study (as the following formula 3).

Innovation-decision period to adopt MNP = {(decide time point – heard about time point)+[heard about time point-(-9)]} + [(feed time point - receive time point)+(receive time point - 6)] = decide time point + feed time point +3 (3)

**REFERENCES:**

1. National Health Commission, All-China Women’s Federation. Chidren nutrition improvement program in poor rural China. 2013 November 19, 2019]; Available from: http://www.gov.cn/gzdt/2013-11/29/content_2538706.htm.
2. National Health Commission, The Ministry of Finance, National Administration of Traditional Chinese Medicine, National Administration of Traditional Chinese Medicine, Notice on Preparing to The Basic Public Health Service in 2019. 2019.

| **Appendix Table S2.** The decision process to adopt MNP among multi-ethnic caregivers | | | | |
| --- | --- | --- | --- | --- |
| **The decision process to adopt MNP** | **N** | **Mean** | **SD** | ***p*-value** ^a^ |
| **Hear about MNP for the first time (child age, months)** ^b^ |  |  |  | **<0.001** |
| Total | 570 | 0.42 | 14.05 |  |
| Han ethnic group | 226 | -1.97 | 15.84 |  |
| Tibetan group | 143 | -0.83 | 15.36 |  |
| Yi ethnic group | 201 | 3.99 | 9.56 |  |
| **Decide to use MNP for the first time (child age, months)** ^c^ |  |  |  | **<0.001** |
| Total | 570 | 7.76 | 4.83 |  |
| Han ethnic group | 226 | 7.12 | 4.67 |  |
| Tibetan group | 143 | 7.23 | 4.40 |  |
| Yi ethnic group | 201 | 8.84 | 5.12 |  |
| **Receive MNP for the first time (child age, months)** ^d^ |  |  |  | **<0.001** |
| Total | 570 | 8.29 | 3.90 |  |
| Han ethnic group | 226 | 7.76 | 3.75 |  |
| Tibetan group | 143 | 7.97 | 3.34 |  |
| Yi ethnic group | 201 | 9.13 | 4.29 |  |
| **Feed the child MNP for the first time (child age, months)** ^e^ |  |  |  | **0.01** |
| Total | 570 | 8.88 | 4.32 |  |
| Han ethnic group | 226 | 8.42 | 4.33 |  |
| Tibetan group | 143 | 8.45 | 3.79 |  |
| Yi ethnic group | 201 | 9.70 | 4.57 |  |
| **Decision period to adopt MNP (duration, months)** ^f^ |  |  |  | **<0.001** |
| Total | 570 | 19.63 | 8.71 |  |
| Han ethnic group | 226 | 18.54 | 8.54 |  |
| Tibetan group | 143 | 18.68 | 7.59 |  |
| Yi ethnic group | 201 | 21.54 | 9.35 |  |
| ^a.^ Group differences of the sample’s continuous variables were analyzed. Boldface indicates statistical significance (*p*<0.05). ^b.^ “Hear about MNP for the first time” was measured by the question: “When did you hear about MNP for the first time?”  ^c.^ “Decide to use MNP for the first time” was measured by the question: “When did you decide to use MNP for the first time?”  ^d.^ “Receive MNP for the first time” was measured by the question: “When did you receive MNP for the first time?”  ^e.^ “Feed the child MNP for the first time” was measured by the question: “When did you feed your child MNP for the first time?”  ^f.^ “Decision period to adopt MNP” was calculated based on “hear about MNP for the first time”, “decide to use MNP for the first time”, “receive MNP for the first time”, and “feed the child MNP for the first time”. | | | | |

| **Appendix Table S3.** Univariate analyze of MNP adopter category among caregivers | | | | | | | |
| --- | --- | --- | --- | --- | --- | --- | --- |
| **Characteristics** | **Total** | **Leaders** | **Followers** | | **Loungers** | **Laggards** | ***p*-value** ^a^ |
| **Caregiver's characteristics** |  |  |  | |  |  |  |
| Age (years) | 38.40 | 37.90 | 39.00 | | 37.50 | 39.00 | 0.67 |
| Female (%) | 90.88 | 93.10 | 91.84 | | 91.77 | 86.24 | 0.33 |
| Ethnic background (%) |  |  |  | |  |  | **0.01** |
| Han | 39.47 | 55.17 | 43.88 | | 40.26 | 26.61 |  |
| Tibetan | 25.26 | 20.69 | 25.51 | | 26.84 | 21.10 |  |
| Yi | 35.26 | 24.14 | 30.61 | | 32.90 | 52.29 |  |
| Education level (%) |  |  |  | |  |  | **0.01** |
| Did not complete primary school | 57.37 | 34.48 | 56.63 | | 54.55 | 70.64 |  |
| Primary school | 12.98 | 13.79 | 14.80 | | 12.55 | 11.01 |  |
| Junior school | 16.32 | 20.69 | 17.86 | | 18.61 | 8.26 |  |
| High school or above | 13.33 | 31.03 | 10.71 | | 14.29 | 10.09 |  |
| Occupation (%) |  |  |  | |  |  | 0.19 |
| Stay-at-home caregivers | 48.07 | 51.72 | 48.98 | | 48.48 | 44.95 |  |
| Farmer/nomadic herder | 30.53 | 27.59 | 30.10 | | 26.41 | 40.37 |  |
| Other ^b^ | 21.40 | 20.69 | 20.92 | | 25.11 | 14.68 |  |
| Household assets level (%) ^c^ |  |  |  | |  |  | **0.01** |
| Low level | 22.63 | 13.79 | 17.86 | | 22.08 | 34.86 |  |
| Lower than mean | 28.25 | 20.69 | 24.49 | | 30.74 | 32.11 |  |
| Higher than mean | 33.16 | 37.93 | 38.78 | | 31.60 | 25.69 |  |
| High level | 15.96 | 27.59 | 18.88 | | 15.58 | 7.34 |  |
| **Personality of caregivers** ^d^ |  |  |  |  | |  |  |
| Extraversion (%) |  |  |  |  | |  | 0.07 |
| Low level | 20.35 | 17.24 | 16.33 | 24.68 | | 19.27 |  |
| Lower than mean | 30.88 | 51.72 | 30.10 | 26.84 | | 33.94 |  |
| Higher than mean | 23.16 | 17.24 | 21.94 | 23.81 | | 25.69 |  |
| High level | 25.61 | 13.79 | 31.63 | 24.68 | | 21.10 |  |
| Agreeableness (%) |  |  |  |  | |  | 0.18 |
| Low level | 24.91 | 24.14 | 19.39 | 27.71 | | 30.28 |  |
| Lower than mean | 25.09 | 37.93 | 26.53 | 22.08 | | 24.77 |  |
| Higher than mean | 33.16 | 27.59 | 35.71 | 35.06 | | 24.77 |  |
| High level | 16.84 | 10.34 | 18.37 | 15.15 | | 20.18 |  |
| Conscientiousness (%) |  |  |  |  | |  | 0.17 |
| Low level | 18.07 | 24.14 | 13.78 | 19.91 | | 21.10 |  |
| Lower than mean | 38.60 | 41.38 | 41.84 | 36.80 | | 36.70 |  |
| Higher than mean | 26.67 | 17.24 | 22.96 | 30.74 | | 25.69 |  |
| High level | 16.67 | 17.24 | 21.43 | 12.55 | | 16.51 |  |
| Emotional stability (%) |  |  |  |  | |  | 0.79 |
| Low level | 24.39 | 31.03 | 22.45 | 25.11 | | 25.69 |  |
| Lower than mean | 32.11 | 24.14 | 30.10 | 34.63 | | 33.03 |  |
| Higher than mean | 25.96 | 27.59 | 26.02 | 25.97 | | 23.85 |  |
| High level | 17.54 | 17.24 | 21.43 | 14.29 | | 17.43 |  |
| Openness to experience (%) |  |  |  |  | |  | 0.60 |
| Low level | 15.61 | 20.69 | 12.24 | 17.32 | | 16.51 |  |
| Lower than mean | 37.72 | 34.48 | 35.71 | 37.66 | | 43.12 |  |
| Higher than mean | 23.51 | 20.69 | 25.00 | 24.68 | | 18.35 |  |
| High level | 23.16 | 24.14 | 27.04 | 20.35 | | 22.02 |  |
| **Child's characteristics** |  |  |  | |  |  |  |
| Age (months) | 14.20 | 14.20 | 14.00 | | 13.70 | 15.70 | **0.01** |
| Boy (%) | 52.28 | 51.72 | 54.59 | | 51.95 | 48.62 | 0.80 |
| Preterm birth (%) ^e^ | 6.41 | 10.71 | 4.21 | | 7.31 | 7.62 | 0.40 |
| Low birth weight (%) ^f^ | 14.01 | 10.34 | 11.98 | | 14.78 | 17.59 | 0.53 |
| Self-reported child health status after birth, average score ^g^ | 0.7 | 0.4 | 0.8 | | 0.7 | 0.7 | 0.20 |

| **Continued Appendix Table S3.** | | | | | | |
| --- | --- | --- | --- | --- | --- | --- |
| **Characteristics** | **Total** | **Leaders** | **Followers** | **Loungers** | **Laggards** | ***p*-value** ^a^ |
| **Cognition and self-efficacy regarding MNP** |  |  |  |  |  |  |
| Have knowledge of MNP component (%) | 11.58 | 27.59 | 12.24 | 12.55 | 4.59 | **0.01** |
| Have knowledge of MNP effect (%) | 27.19 | 44.83 | 26.53 | 27.27 | 21.10 | 0.08 |
| Have knowledge of MNP feeding method (%) | 56.49 | 72.41 | 64.29 | 53.68 | 43.12 | **<0.001** |
| Susceptibility of child anemia (%) |  |  |  |  |  | **0.04** |
| 4Not at all | 41.75 | 37.93 | 39.80 | 38.53 | 53.21 |  |
| Probably | 35.44 | 48.28 | 40.31 | 35.93 | 23.85 |  |
| Definitely | 22.81 | 13.79 | 19.90 | 25.54 | 22.94 |  |
| Severity of child anemia (%) |  |  |  |  |  | 0.22 |
| Not at all | 59.47 | 55.17 | 55.10 | 59.31 | 68.81 |  |
| Probably | 18.07 | 24.14 | 18.37 | 20.35 | 11.93 |  |
| Definitely | 22.46 | 20.69 | 26.53 | 20.35 | 19.27 |  |
| Self-efficacy in adopting MNP (%) |  |  |  |  |  | **0.01** |
| Not at all | 26.32 | 17.24 | 23.47 | 24.68 | 38.53 |  |
| Probably not | 10.53 | 0.00 | 7.65 | 13.42 | 11.93 |  |
| Neutral | 15.79 | 10.34 | 14.80 | 15.58 | 19.27 |  |
| Probably | 28.25 | 51.72 | 31.63 | 28.57 | 15.60 |  |
| Definitely | 19.12 | 20.69 | 22.45 | 17.75 | 14.68 |  |
| **Communication channel and message regarding MNP for the first time** |  |  |  |  |  |  |
| Communication channel for the first time (%) |  |  |  |  |  | 0.25 |
| Family members or relatives | 9.65 | 10.34 | 8.16 | 9.96 | 11.93 |  |
| Villagers | 8.42 | 13.79 | 8.16 | 8.66 | 7.34 |  |
| Village doctors | 54.91 | 51.72 | 48.47 | 58.01 | 58.72 |  |
| Township doctors | 27.02 | 24.14 | 35.20 | 23.38 | 22.02 |  |
| Communication message for the first time (%) |  |  |  |  |  | **0.02** |
| MNP was free | 32.63 | 31.03 | 32.14 | 31.60 | 34.86 |  |
| Health benefits of MNP | 58.42 | 41.38 | 58.16 | 61.90 | 57.80 |  |
| MNP feeding methods | 8.95 | 27.59 | 9.69 | 6.49 | 7.34 |  |
| **Delivery pattern and distance of MNP** |  |  |  |  |  |  |
| Delivery pattern regarding MNP |  |  |  |  |  | 0.20 |
| Taking MNP from township health center | 52.11 | 55.17 | 54.59 | 54.55 | 42.20 |  |
| Taking MNP from village clinics | 33.86 | 31.03 | 29.59 | 32.90 | 44.95 |  |
| The health personnel or village cadre delivered MNP to home | 14.04 | 13.79 | 15.82 | 12.55 | 12.84 |  |
| Distance to supply site (km) | 4.20 | 4.60 | 3.90 | 4.10 | 4.80 | 0.40 |
| ^a.^ Chi-square tests of independence between ethnic groups were conducted. Boldface indicates statistical significance (*p*<0.05). ^b.^ Other occupations include off-farm part-time jobs and self-employment. ^c.^ A household asset index was calculated using polychoric principal components analysis[20, 21] based on whether the household owned or had access to a water heater, washing machine, refrigerator, air conditioner, television, computer, motorcycle, car or truck.  ^d.^ The Big Five personality traits scale, developed from the 1980s onwards in psychological trait theory, was applied to test the personality of caregivers in our study. The scale identified five personality dimensions: extraversion, agreeableness, openness to experience, conscientiousness, and emotional stability.  ^e.^ Preterm birth means the infant was born alive before 37 weeks of pregnancy duration.[22] ^f.^ Low birth weight means the birth weight was lower than 2500 grams.[23]  ^g.^ Caregivers were asked about child health status after birth, the responses were rated on a 5-point Likert scale that ranged from 1 for ‘unhealthy’ to 5 for ‘healthy’. | | | | | | |

| **Appendix Table S4.** Ordered logistic regression model of the associated factors of MNP adopter category in Han, Tibetan, and Yi ethnic groups | | | | | | | | | | | |
| --- | --- | --- | --- | --- | --- | --- | --- | --- | --- | --- | --- |
| **Characteristics** | **MNP adopter category** ^a,b^ | | | | | | | | | | |
|  | **AOR** | **95% CI** | |  | **AOR** | **95% CI** | |  | **AOR** | **95% CI** | |
| **Caregiver’s characteristics** |  |  |  |  |  |  |  |  |  |  |  |
| Education level | 0.84 | 0.68 | 1.04 |  | 1.13 | 0.84 | 1.52 |  | 1.05 | 0.77 | 1.42 |
| **The personality of caregivers** ^c^ |  |  |  |  |  |  |  |  |  |  |  |
| Extraversion | 0.92 | 0.73 | 1.15 |  | 1.17 | 0.9 | 1.53 |  | 0.84 | 0.68 | 1.03 |
| **Cognition and self-efficacy regarding MNP** |  |  |  |  |  |  |  |  |  |  |  |
| Knowledge regarding the MNP component | 0.90 | 0.41 | 1.98 |  | 0.61 | 0.16 | 2.27 |  | 1.05 | 0.22 | 4.87 |
| Knowledge regarding the MNP effect | 0.77 | 0.39 | 1.49 |  | 1.36 | 0.67 | 2.77 |  | 0.91 | 0.47 | 1.76 |
| Knowledge regarding the MNP feeding method | 0.55 | 0.3 | 1 |  | 1.01 | 0.6 | 1.71 |  | 0.79 | 0.44 | 1.41 |
| Susceptibility perception regarding childhood anemia | **1.58**** | **1.18** | **2.12** |  | 1.12 | 0.71 | 1.74 |  | 0.86 | 0.66 | 1.13 |
| Self-efficacy in adopting MNP | **0.74***** | **0.64** | **0.85** |  | 0.93 | 0.7 | 1.25 |  | 0.84 | 0.66 | 1.06 |
| **Communication channel and message regarding MNP for the first time** |  |  |  |  |  |  |  |  |  |  |  |
| Family members or relatives*free | Ref. | Ref. | Ref. |  | Ref. | Ref. | Ref. |  | Ref. | Ref. | Ref. |
| Family members or relatives*benefit | 0.59 | 0.13 | 2.76 |  | 0.66 | 0.1 | 4.39 |  | - | - | - |
| Family members or relatives*method | 1.50 | 0.19 | 11.55 |  | - | - | - |  | 0.18 | 0.02 | 1.4 |
| Villagers*free | **0.36*** | **0.14** | **0.96** |  | - | - | - |  | 0.98 | 0.12 | 7.77 |
| Villagers*benefit | 0.44 | 0.09 | 2.24 |  | 1.55 | 0.21 | 11.51 |  | 2.38 | 0.22 | 25.54 |
| Villagers*method | - | - | - |  | 1.71 | 0.53 | 5.46 |  | **0.07**** | **0.01** | **0.49** |
| Village doctors*free | 0.72 | 0.16 | 3.17 |  | 0.5 | 0.11 | 2.25 |  | 1.36 | 0.15 | 12.46 |
| Village doctors*benefit | 0.59 | 0.14 | 2.46 |  | 1.22 | 0.44 | 3.36 |  | 0.75 | 0.09 | 5.94 |
| Village doctors*method | 1.19 | 0.01 | 168.13 |  | 0.44 | 0.08 | 2.51 |  | 0.45 | 0.04 | 5.8 |
| Township doctors*free | 0.42 | 0.13 | 1.38 |  | 1.57 | 0.44 | 5.53 |  | 0.69 | 0.07 | 6.63 |
| Township doctors*benefit | 0.39 | 0.1 | 1.56 |  | 0.63 | 0.17 | 2.31 |  | 2.48 | 0.3 | 20.41 |
| Township doctors*method | **0.12*** | **0.02** | **0.72** |  | **0.22*** | **0.06** | **0.86** |  | - | - | - |
| Note: AOR=adjusted odds ratio; CI=confidence interval. Boldface indicates statistical significance (*p<0.05, **p<0.01, ***p<0.001). The dash "-" indicates that caregivers did not report the type of communication, for example, in the Han group, no caregivers received information on MNP feeding methods from villagers.  a. In the ordered logistic regression model, the MNP adopter category was regarded as the outcome variable, the value assignment of MNP adopter category was: 1=leaders; 2=followers; 3=loungers; 4=laggards.  b. In the ordered logistic regression model, the p-value of variables less than 0.10 from univariate analysis were enrolled as the independent variables, including education level, the personality of extraversion, knowledge regarding MNP component, knowledge regarding MNP effect, knowledge regarding MNP method, susceptibility of child anemia, have confidence in solving all problems on adopting MNP, and interaction between communication channel and message regarding MNP for the first time.  c. The Big Five personality traits scale, developed from the 1980s onwards in psychological trait theory, was applied to test the personality of caregivers in our study. The scale identified five personality dimensions: extraversion, agreeableness, openness to experience, conscientiousness, and emotional stability. | | | | | | | | | | | |
